# Clinical deterioration during antituberculosis treatment in Africa: Incidence, causes and risk factors

**DOI:** 10.1186/1471-2334-10-83

**Published:** 2010-03-30

**Authors:** Dominique J Pepper, Suzaan Marais, Robert J Wilkinson, Feriyl Bhaijee, Gary Maartens, Helen McIlleron, Virginia De Azevedo, Helen Cox, Cheryl McDermid, Simiso Sokhela, Janisha Patel, Graeme Meintjes

**Affiliations:** 1 Infectious Diseases Unit, GF Jooste Hospital, Cape Town, South Africa; 2 Institute of Infectious Diseases and Molecular Medicine, University of Cape Town, South Africa; 3 Department of Medicine, University of Cape Town, South Africa; 4 Division of Medicine, Imperial College of London, London, UK; 5 MRC National Institute for Medical Research, Mill Hill, London, NW7 1AA, UK; 6 Groote Schuur Hospital, Cape Town, South Africa; 7 Division of Clinical Pharmacology, Department of Medicine, University of Cape Town, South Africa; 8 Khayelitsha Site B Tuberculosis Clinic, City Health, Cape Town, South Africa; 9 Macfarlane Burnet Institute for Medical Research and Public Health, Australia; 10 MedecinsSans Frontieres, Cape Town, South Africa

## Abstract

**Background:**

HIV-1 and *Mycobacterium tuberculosis *cause substantial morbidity and mortality. Despite the availability of antiretroviral and antituberculosis treatment in Africa, clinical deterioration during antituberculosis treatment remains a frequent reason for hospital admission. We therefore determined the incidence, causes and risk factors for clinical deterioration.

**Methods:**

Prospective cohort study of 292 adults who initiated antituberculosis treatment during a 3-month period. We evaluated those with clinical deterioration over the following 24 weeks of treatment.

**Results:**

Seventy-one percent (209/292) of patients were HIV-1 infected (median CD4+: 129 cells/μL [IQR:62-277]). At tuberculosis diagnosis, 23% (34/145) of HIV-1 infected patients qualifying for antiretroviral treatment (ART) were receiving ART; 6 months later, 75% (109/145) had received ART. Within 24 weeks of initiating antituberculosis treatment, 40% (117/292) of patients experienced clinical deterioration due to co-morbid illness (n = 70), tuberculosis related illness (n = 47), non AIDS-defining HIV-1 related infection (n = 25) and AIDS-defining illness (n = 21). Using HIV-1 uninfected patients as the referent group, HIV-1 infected patients had an increasing risk of clinical deterioration as CD4+ counts decreased [CD4+>350 cells/μL: RR = 1.4, 95% CI = 0.7-2.9; CD4+:200-350 cells/μL: RR = 2.0, 95% CI = 1.1-3.6; CD4+<200 cells/μL: RR = 3.0, 95% CI = 1.9-4.7]. During follow-up, 26% (30/117) of patients with clinical deterioration required hospital admission and 15% (17/117) died. Fifteen deaths were in HIV-1 infected patients with a CD4+<200 cells/μL.

**Conclusions:**

In multivariate analysis, HIV-1 infection and a low CD4+ count at tuberculosis diagnosis were significant risk factors for clinical deterioration and death. The initiation of ART at a CD4+ count of <350 cells/μL will likely reduce the high burden of clinical deterioration.

## Background

Adherence to antituberculosis treatment in advanced human immunodeficiency virus type 1 (HIV-1) infection results in rapid sterilisation of sputum, radiographic improvement and a low risk of relapse [1]. The benefits of antiretroviral treatment (ART) in reducing HIV-1 replication and restoring pathogen-specific immunity are well described [2,3]. Despite the availability of antituberculosis and antiretroviral treatment in Africa, clinical deterioration during antituberculosis treatment in HIV-1 infected patients remains an important reason for hospital admission and death [4,5].

Profoundly immune-suppressed HIV-1 infected patients may encounter a complicated clinical course after starting antituberculosis treatment. While initiation of ART during antituberculosis treatment reduces mortality [6], the optimal interval from antituberculosis treatment to initiation of ART is not known. Early initiation of ART restores pathogen-specific immunity, but also significantly increases the risk of the tuberculosis-associated immune reconstitution inflammatory syndrome (TB-IRIS) [7]. Conversely, a delay in initiation of ART may allow additional AIDS-defining illnesses to manifest. Other reasons for clinical deterioration during antituberculosis treatment include antimicrobial resistance, suboptimal antituberculosis drug concentrations, drug reactions, and other opportunistic illnesses [8,9].

The greatest burden of HIV-1 infection and tuberculosis occurs in resource-limited settings, such as South Africa, where health systems are overwhelmed and rapid diagnostic tools are not readily available. In 2006, 337,400 of 482,000 tuberculosis patients in South Africa were HIV-1 co-infected and 105,000 tuberculosis deaths were reported [10]. The fatal consequences of HIV-1/*Mycobacterium tuberculosis *co-infection are well described [11,12]. However, the incidence of clinical deterioration during antituberculosis treatment amongst HIV-1 infected patients (compared to HIV-1 uninfected patients) is unknown. Determining the causes and risk factors for clinical deterioration during antituberculosis treatment may inform initiatives to reduce the burden on both tuberculosis and ART programmes.

In this study, we assessed patients at initiation of antituberculosis treatment and followed them for 24 weeks, in order to determine the incidence, causes and risk factors for clinical deterioration. We also discuss initiatives to reduce the high burden of clinical deterioration in resource-limited settings.

## Methods

We conducted a prospective cohort study at Khayelitsha Site B tuberculosis clinic (Cape Town, South Africa) from 1 June 2008 through 15 February 2009. We assessed adult (≥ 18 years age) patients diagnosed with tuberculosis at Khayelitsha Site B tuberculosis clinic from 1 June 2008 through 31 August 2008 (3-month assessment period). Informed consent was obtained from all enrolled patients. HIV-1 voluntary counselling and testing is offered to all patients diagnosed with tuberculosis at Site B Khayelitsha. Patients were followed for 24 weeks from initiation of antituberculosis treatment. Our study was nested within a tuberculosis drug susceptibility testing (DST) survey in which first-line DST (for isoniazid and rifampin) was routinely performed, regardless of HIV-1 status or previous tuberculosis. The Research Ethics Committee of the University of Cape Town approved this study (REC 178/2008).

### Study site and setting

Khayelitsha Site B tuberculosis clinic is a primary-level outpatient health care facility, which serves ~100,000 people within its high-density, low-income catchment area. Approximately 1,200 adult cases of tuberculosis are diagnosed per annum at Site B tuberculosis clinic. In 2006, the antenatal HIV-1 seroprevalence in this community was 33% (95%: 29.1 - 36.9%) [13]. According to national protocol, patients with a new diagnosis of tuberculosis receive 6 months of daily antituberculosis treatment (isoniazid, rifampin, pyrazinamide, and ethambutol for 2 months, followed by isoniazid and rifampin for 4 months) [14]. The retreatment regimen is: isoniazid, rifampin, pyrazinamide, ethambutol, and streptomycin for 2 months, followed by isoniazid, rifampin, pyrazinamide, and ethambutol for 1 month, and then isoniazid, rifampin, and ethambutol for 5 months. First-line ART in South Africa is stavudine, lamivudine, and either nevirapine or efavirenz. Efavirenz is preferred for patients who are receiving rifampin-based antituberculosis treatment. Patients with a CD4+ cell count <200 cells/μL and/or a history of a World Health Organization (WHO) stage 4 illness, other than extra-pulmonary tuberculosis, are eligible to commence ART [15]; ART is typically commenced 2 months after initiation of antituberculosis treatment. If the CD4+ cell count is <50 cells/μL or a serious HIV-1-related illness is present, ART may be commenced 2 weeks after starting antituberculosis treatment [15]. To date, >5,000 people have initiated ART at Khayelitsha Site B HIV clinic.

### Definitions

***"***Tuberculosis patients" refers to patients diagnosed with tuberculosis and initiated on antituberculosis treatment. We defined microbiologically-confirmed tuberculosis as *Mycobacterium tuberculosis *cultured or acid-fast bacilli visualised in a biological specimen. Biological specimens included sputum, pleural fluid, urine, nodal aspirates, pericardial aspirates and cerebrospinal fluid. We defined microbiologically-unconfirmed tuberculosis according to the WHO case definitions for smear-negative and extra-pulmonary tuberculosis [16]. Biological specimens were obtained at tuberculosis diagnosis, at 8 and 20 weeks' follow-up and at clinical deterioration. First-line DST using the GenoType MTBDRplus assay [17] was performed on biological specimens that were smear-positive for acid-fast bacilli and/or culture positive for *Mycobacterium tuberculosis*. We defined multidrug-resistant (MDR) tuberculosis as *M. tuberculosis *resistant to isoniazid and rifampin.

We defined clinical deterioration as symptomatic worsening or failure to stabilise within 24 weeks following initiation of antituberculosis treatment. We subdivided the causes of clinical deterioration into HIV-1 related illnesses and HIV-1 unrelated illnesses. HIV-1 related causes included AIDS defining illnesses (according to WHO stage 4 criteria [18]), and non AIDS-defining illnesses i.e. Non-AIDS defining HIV-1 related infections. HIV-1 unrelated illnesses included tuberculosis related illnesses, and illnesses unrelated to tuberculosis i.e. co-morbid illnesses. Tuberculosis-related illnesses included MDR-TB, deterioration due to poor adherence, paradoxical tuberculosis-associated immune reconstitution inflammatory syndrome (TB-IRIS) and paradoxical reactions. A co-morbid illness was an acute illness that occurred after tuberculosis diagnosis and was considered not to be directly attributable to HIV-1 or tuberculosis. A co-morbid illness also included an acute deterioration of a pre-existing condition. For example, we regarded diabetic ketoacidosis in a diabetic patient as a co-morbid illness.

We defined paradoxical TB-IRIS according to the consensus clinical case definition for resource-limited settings [7]. We defined paradoxical reactions using an adaptation of this consensus clinical case definition of paradoxical TB-IRIS [7]. Three criteria were required for a diagnosis of paradoxical reaction:

1. Diagnosis of tuberculosis (microbiologic confirmation or according to WHO criteria [16]) with initial response to antituberculosis treatment,

2. The recurrence/new onset of tuberculosis disease manifestations within 24 weeks of antituberculosis treatment, and

3. Exclusion of alternative explanations for clinical deterioration (such as antituberculosis drug resistance, poor adherence, drug toxicity or reaction, or an additional infection).

Paradoxical reactions were diagnosed in both HIV-1 infected and uninfected patients. HIV-1 infected patients receiving ART at clinical deterioration were diagnosed with paradoxical TB-IRIS rather than a paradoxical reaction according to our definitions.

We defined a patient as being lost to follow-up if we were unable to trace a patient 24 weeks after initiation of antituberculosis treatment. We used clinic and hospital medical notes, as well as the National Health Laboratories Service database to trace specimens and the Provincial Government of the Western Cape's electronic tuberculosis register to trace patients.

### Assessment of patients with clinical deterioration

The study was discussed with physicians and nurses at the tuberculosis clinic prior to commencement of the study. Adult patients with clinical deterioration within 24 weeks of initiation of antituberculosis treatment were prospectively evaluated (regardless of severity of illness, eventual diagnosis, or inpatient or outpatient management) to determine the reason for clinical deterioration. Stable patients were assessed at Khayelitsha Site B tuberculosis clinic; patients requiring hospital admission or invasive outpatient procedures were assessed at GF Jooste Hospital (the clinic's referral hospital). At clinical deterioration, we routinely obtained biological specimens to investigate drug-resistant *M. tuberculosis*. Specimens were also cultured for bacterial organisms.

### Data collection and analysis

Data collected included demographic information, tuberculosis specimen results, HIV-1 status, antiretroviral treatment, CD4+ cell count at tuberculosis diagnosis, diagnosis at clinical deterioration and outcome 24 weeks after initiation of antituberculosis treatment. Statistical analyses were performed using Stata 10.0 (Texas, USA). Wilcoxon rank-sum and Kruskall-Wallis tests were used for group comparisons, and Fisher's exact tests for proportion comparisons. Variables with the outcome of interest were entered into Cox proportional hazards models to assess the independent effects of covariates. We censored patients at clinical deterioration when performing Cox-proportional hazards model analysis and relative risk calculations. Significant variables were removed from the model to assess whether these effects remained. The assumptions of the Cox model were verified; non-informative censoring was performed and the tests for the proportional hazards assumption were not significant. We censored patients at loss to follow-up when determining the incidence rate of clinical deterioration (illnesses diagnosed per 100 months of follow-up). Individual patients could contribute more than one event in the calculation of the incidence rate.

## Results

**Figure 1:**
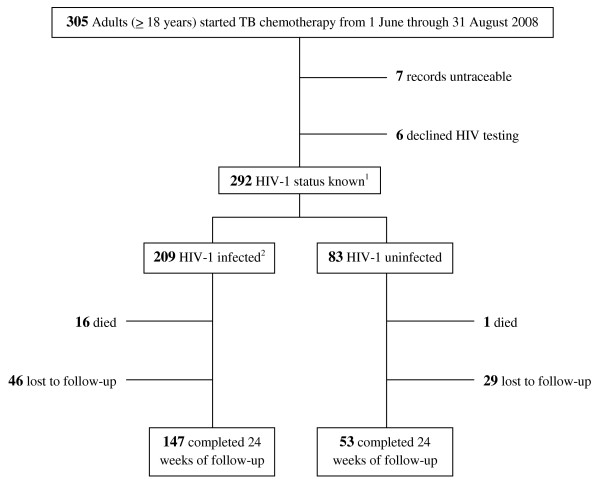
**Flow-diagram of 305 adult patients who started antituberculosis treatment from 1 June - 31 August 2008**. ^1^Subsequent data analysis for 292 patients with known HIV-1 status ^2^CD4 counts not performed in 3 of 209 HIV-1 infected patients

During the 3-month assessment period (figure 1), 305 adults (≥ 18 years age) initiated antituberculosis treatment, 7 of whom had untraceable clinical records and 6 of whom declined HIV-1 testing. We restricted our data analysis to the 292 (96%) patients whose HIV-1 status and clinic records were available. In 209 HIV-1 infected and 83 HIV-1 uninfected patients, loss to follow-up (46 [22%] of 209 vs. 29 [35%] of 83, p-value = 0.026) and mortality (16 [8%] of 209 vs. 1 [1%] of 83, p-value = 0.048) differed significantly at 24 weeks.

At initial tuberculosis diagnosis (table 1), HIV-1 infected patients were more likely than HIV-1 uninfected patients to be female, be of younger age, have extra-pulmonary tuberculosis and be diagnosed with tuberculosis at the referral hospital. HIV-1 uninfected patients were more likely than HIV-1 infected patients to have microbiologic confirmation of tuberculosis at initial tuberculosis diagnosis and during the 24 weeks of follow-up.

**Table 1:** Baseline characteristics and microbiologic confirmation of tuberculosis in 209 HIV-1 infected and 83 HIV-1 uninfected patients receiving antituberculosis treatment

	HIV-1 infected (n = 209)	HIV-1 uninfected (n = 83)	p-value
**Female, n (%)**	112 (54)	25 (30)	<0.001

**Age in years, median (IQR)**	35 (30-41)	38 (29-49)	0.035

**CD4 count at TB diagnosis, n (%):**			
**>350 cells/mm**^3^	36 (17)	-	
**200 - 350 cells/mm**^3^	37 (18)	-	
**< 200 cells/mm**^3^	133 (64)	-	
**Not performed**	3 (1)	-	

**Previous TB, n (%)**	71 (34)	19 (23)	0.070

**TB diagnosed at referral hospital, n (%):**	79 (38)	13 (16)	<0.001

**Microbiologic confirmation at TB diagnosis**^1^**, n (%)**	123 (59)	68 (82)	<0.001

**Drug susceptibilities known at TB diagnosis**^1^**, n (%)**	67 (32)	49 (59)	<0.001

**Culture sent at TB diagnosis, n (%)**	169 (81)	68 (82)	1.000

**Extra-pulmonary TB, n (%):**	82 (39)	14 (17)	<0.001

**Weight at TB diagnosis in kg, median (IQR)**	54 (49-62)	54 (49-64)	0.644

**Microbiologic confirmation at TB diagnosis and during 24 weeks of follow-up**^2^**, n (%)**	153 (73)	70 (84)	0.048

**Drug susceptibilities known at TB diagnosis and during 24 weeks of follow-up**^2^**, n (%)**	117 (56)	57 (69)	0.296

**Culture sent at TB diagnosis and/or during 24 weeks of follow-up, n (%)**	190 (91)	76 (92)	1.000

Key:

^1 ^within 14 days of TB diagnosis. No TB specimens sent in 12 HIV-1 infected patients and 3 HIV-1 uninfected patients at TB diagnosis.

^2 ^no TB specimens sent in 2 HIV-1 infected patients.

TB = tuberculosis, IQR = inter-quartile range, OR = odds-ratio, 95% CI = 95% confidence interval

Prior to tuberculosis diagnosis, 34 (23%) of 145 HIV-1 infected patients who qualified for ART under national guidelines were receiving ART. Six months later, 109 (75%) of 145 patients had received ART.

### Causes of Clinical Deterioration and Hospital Admission

During the 24 weeks of follow-up, 117 (40%, 95% CI: 35-46%) of 292 tuberculosis patients experienced clinical deterioration, of whom 101 were HIV-1 infected and 16 were HIV-1 uninfected. Causes of clinical deterioration (table 2) included: co-morbid illnesses (70 patients), tuberculosis-related illnesses (47 patients), non AIDS-defining HIV-1 related infections (25 patients) and AIDS-defining illnesses (21 patients). Peripheral neuropathy, enteric illness and deep venous thrombosis were frequent co-morbid illnesses. TB-IRIS and paradoxical reactions were frequent tuberculosis-related illnesses. Oesophageal candida, *Pneumocystis jirovecii *pneumonia and cryptococcal meningitis were frequent AIDS-defining illnesses. Of 117 patients who experienced deterioration, 30 (26%) required hospital admission [27 (27%) of 101 HIV infected and 3 (19%) of 16 HIV-1 uninfected patients (p-value = 0.756)]. Causes of inpatient hospital admission were paradoxical reaction or TB-IRIS (9 patients), new AIDS-defining illness (8 patients), deep venous thrombosis (6 patients), MDR-TB (2 patients), cardiomyopathy (1 patient), pneumothorax (1 patient), symptomatic deterioration due to poor adherence with antituberculosis treatment (1 patient), hyperglycaemic emergency (1 patient) and seizure disorder (1 patient).

**Table 2:** Illnesses (n = 199) in 101 HIV-1 infected and 16 HIV-1 uninfected patients who experienced clinical deterioration during 24 weeks of antituberculosis treatment

	HIV-1 infected	HIV-1 uninfected
number of illnesses = n, (total number of patients = pnts)	**180 (101)**	**19 (16)**

**1. Tuberculosis related illnesses**, n (pnts)	**49 (43)**	**5 (5)**
TB-IRIS	22	-
Paradoxical reaction	17	4
MDR-TB^1^	4	1
Deterioration due to poor adherence	5	-
Low rifampin concentration in comparison to the recommended range	1	-

**2. AIDS-defining illnesses**, n (pnts)	**29 (21)**	**-**
Oesophageal candida	11	-
*Pneumocystis jiroveci *pneumonia^2^	5	-
Cryptococcal meningitis	4	-
Cytomegalovirus retinitis	3	-
Other^3^	6	-

**3. Non-AIDS defining HIV-1 related infections**, n (pnts)	**27 (25)**	**-**
Superficial herpes infection^4^	9	-
Bacterial infection^5^	9	-
Fungal (oral/vaginal/superficial) infection	9	-

**4. Co-morbid illnesses**, n (pnts)	**76 (59)**^6^	**14 (11)**^7^

Key:

TB = tuberculosis; TB-IRIS = TB-associated immune reconstitution inflammatory syndrome

^1 ^MDR-TB: *Mycobacterium tuberculosis *resistant to rifampin and isoniazid

^2^All 5 patients diagnosed with Pneumocystis jirovecii pneumonia received trimethoprim sulfamethoxazole chemoprophylaxis (160/800 mg daily).

^3 ^Other = HIV-1 associated nephropathy (2), HIV-1 associated encephalopathy (2), Disseminated Kaposi's sarcoma (2)

^4 ^5 patients diagnosed with herpes simplex, 4 patients diagnosed with herpes zoster (all < 1 month duration)

^5 ^Urinary tract infection (3) - *E. coli, Klebsiella sp*., enterococcus; Pelvic inflammatory disease (3); *H. influenza *pneumonia (1); otitis media(1); carbuncle(1)

^6 ^**76 illnesses diagnosed in 59 patients:**

**CD4 > 350 cells/mm**^3 ^= 7 illnesses diagnosed in 7 patients: deep venous thrombosis (2); sacroiliitis (1); acute asthmatic attack (1); cardiomyopathy (1); severe epistaxis (1); supra-condylar fracture (1),

**CD4200 - 350 cells/mm**^3 ^= 12 illnesses diagnosed in 7 patients: enteric illness with no pathogen isolated (4); hyperglycaemic emergency (2); seizures, cause not determined (2); acute asthmatic attack (1); efavirenz side-effect (1); deep venous thrombosis (1); haemorrhoids/fistula-in-ano (1),

**CD4 < 200 cells/mm**^3 ^= 56 illnesses in 44 patients: peripheral neuropathy (12); enteric illness with no pathogen isolated (8); deep venous thrombosis (7); dermatitis (4); cerebrovascular accident (3); scabies (3); minor traumatic injury (3); pneumothorax (2); pancreatitis (1); cardiomyopathy (1); miscarriage (1); cryptococcal IRIS (immune reconstitution inflammatory syndrome) adenitis (1); drug induced hepatitis (1); cause not determined but patient subsequently improved (9).

^7 ^**14 illnesses diagnosed in 11 patients**: Drug side effect (3) - hydrochlorothiazide (1), pyrazinamide (1), kanamycin (1); hyperglycaemic emergency (2); post-TB bronchiectasis (1); peripheral neuropathy (1); pulmonary silicosis (1); miscarriage (1); cause not determined but patient subsequently improved (5)

### Risk Factors for Clinical Deterioration

**Figure 2:**
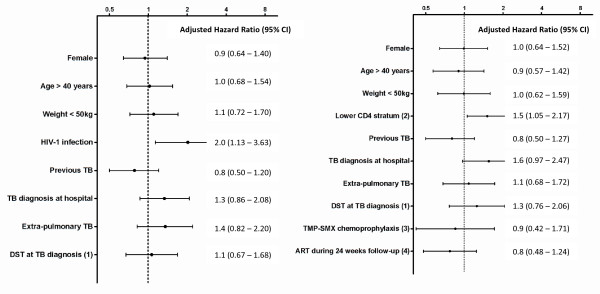
a: Hazard ratios (95% CI) of risk factors for clinical deterioration during 24 weeks of antituberculosis (TB) treatment in 292 patients (Cox proportional hazards model) and Figure 2b>: Hazard ratios (95% CI) of risk factors for clinical deterioration during 24 weeks of TB treatment in 206 HIV-1 infected patients* (Cox proportional hazards model). *CD4 count not performed in 3 of 209 HIV-1 infected patients (1) Microbiologic confirmation with drug susceptibilities known at TB diagnosis (2) CD4 strata used: Stratum 1 = CD4+ > 350 cells/ μL, stratum 2 = CD4+ from 200 – 350 cells/ μL,  stratum 3 = CD4+ < 200 cells/ μL; (3) TMP-SMX chemoprophylaxis = Trimethoprim sulfamethoxazole 160/800mg daily (4) Antiretroviral treatment regimens as follows: D4T/3TC/EFV (89), D4T/3TC/NVP (3),  AZT/3TC/EFV (13), AZT/3TC/NVP (2), TDF/3TC EFV (2); D4T= stavudine 30mg twice daily,  3TC = lamivudine 150mg twice daily, EFV = efavirenz 600mg nocte, AZT = zidovudine 300mg  twice daily, NVP = nevirapine 200mg twice daily, TDF = tenofovir 300mg daily TB = tuberculosis, DST = drug susceptibility testing, ART = antiretroviral treatment, P = p-value  (significant if < 0.05), 95% CI = 95% confidence interval

In the 292 tuberculosis patients, 4 factors were significantly associated with clinical deterioration in univariate analysis: HIV-1 infection, diagnosis of tuberculosis at the referral hospital, evidence of extra-pulmonary tuberculosis, and absence of a DST result at tuberculosis diagnosis. Only HIV-1 infection (figure 2a) remained significant in multivariate analysis (adjusted hazard ratio [aHR]= 2.0, 95% CI = 1.1-3.6).

In subsequent analysis (figures not shown), we assessed whether the probability of clinical deterioration from non-HIV-1 related causes was associated with HIV-1 infection. In univariate analysis, we found a significant association between HIV-1 infection and non-HIV-1 related causes for deterioration (RR = 1.3, 95% CI: 1.07 - 1.50). However, in the Cox proportional hazards model, using the same variables as figure 2a, this significant association was not confirmed (HR = 1.5, 95% CI: 0.81-2.64). This analysis suggests that HIV-1 infection is a significant variable for clinical deterioration because of HIV-1 related illnesses (either AIDS- or non AIDS-defining illnesses).

In the 209 HIV-1 infected tuberculosis patients, 3 factors were significantly associated with clinical deterioration in univariate analysis: a lower CD4+ count, diagnosis of tuberculosis at the referral hospital, and antiretroviral treatment received during antituberculosis treatment. Only a lower CD4+ stratum at tuberculosis diagnosis (figure 2b) remained significant in multivariate analysis (aHR = 1.5, 95% CI = 1.1-2.2).

In subsequent analysis (figures not shown), we assessed whether the probability of clinical deterioration from non-HIV-1 related causes was associated with decreasing CD4+ counts. We did not find a significant association between decreasing CD4+ count strata and non-HIV-1 related causes in both the univariate analysis (P = 0.189) and the Cox proportional hazards model (HR = 1.3, 95%CI: 0.88 - 1.97). This analysis suggests that decreasing CD4+ counts are a significant risk factor for clinical deterioration because of their association with HIV-1 related illnesses (either AIDS- or non AIDS-defining illnesses).

### Relative Risk and Incidence Rate of Clinical Deterioration

HIV-1 infection and a low CD4+ count were the only significant risk factors for clinical deterioration in multivariate analysis. We therefore determined the relative risk and incidence rate of clinical deterioration according to HIV-1 status and CD4+ stratum. HIV-1 infected patients were more likely than HIV-1 uninfected patients to experience clinical deterioration (RR = 2.6, 95%CI: 1.6-4.0). Using HIV-1 uninfected patients as the referent group, the relative risk (RR) of clinical deterioration increased as the CD4+ counts in HIV-1 infected patients decreased (CD4+ >350 cells/μL: RR = 1.4, 95% CI = 0.7-2.9; CD4+200-350 cells/μL: RR = 2.0, 95% CI = 1.1-3.6; CD4+ <200 cells/μL: RR = 3.0, 95% CI = 1.9-4.7). The incidence rate (IR) of clinical deterioration (illnesses diagnosed per 100 months of follow-up) also increased as the CD4+ counts decreased. Incidence rates differed significantly between HIV-1 uninfected patients (IR = 5.1, 95% CI = 3.1-7.5) and HIV-1 infected patients with a CD4+ count of 200-350 cells/μL (IR = 12.3, 95% CI = 8.2-17.0). Similarly, incidence rates differed significantly between HIV-1 infected patients with a CD4+ count of 200-350 cells/μL and HIV-1 infected patients with a CD4+ count < 200 cells/μL (IR = 20.7, 95% CI 17.8-23.9).

**Figure 3:**
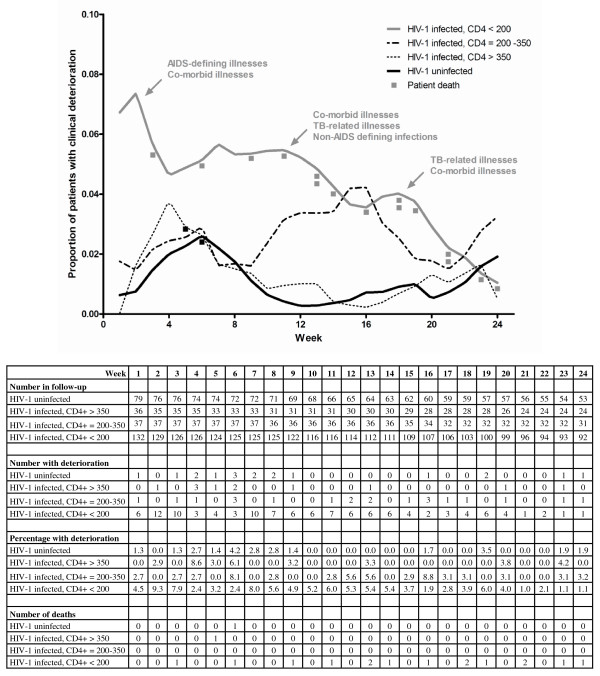
**Lowess plot showing the proportion of patients who experienced clinical deterioration during 24 weeks of antituberculosis treatment**.

Figure 3 is a Lowess plot showing the proportion of patients who experienced clinical deterioration during the 24 weeks of antituberculosis treatment. The initial peak at 6 weeks in HIV-1 uninfected patients corresponds with tuberculosis-related illnesses (mostly paradoxical reactions) and the baseline fluctuations represent co-morbid illnesses. The curve for HIV-1 infected patients with a CD4+ count > 350 cells/μL is similar to that of HIV-1 uninfected patients, despite an earlier peak for paradoxical reactions. The proportion of HIV-1 infected patients with a CD4+ count of 200-350 cells/μL who experienced clinical deterioration substantially increased after 10 weeks of follow-up. Furthermore, a substantially higher proportion of HIV-1 infected patients with a CD4+ count <200 cells/μL experienced clinical deterioration compared to other CD4+ strata.

### Mortality

Fifteen of 17 deaths occurred in HIV-1 infected patients with a CD4+ count < 200 cells/μL. The median interval from antituberculosis treatment to death was 98 days (IQR = 59 -128). Eight of 17 deaths occurred between 10 and 20 weeks of follow-up. Diagnoses at time of death included: AIDS-defining illnesses (5), poor adherence with antituberculosis treatment (4), paradoxical neurologic TB-IRIS (3), enteric illness (2), MDR-TB (1), pulmonary embolus (1) and tension pneumothorax (1).

### Loss to follow-up

Within 24 weeks of commencing antituberculosis treatment, 75 patients were lost to follow-up. The median interval from antituberculosis treatment to loss to follow-up was 82 days (IQR = 48 -125). The CD4+ counts of 46 HIV-1 infected patients who were lost to follow-up (median 150 cells/μL, IQR = 67-347) did not differ significantly (P = 0.362) from the CD4+ counts of 147 HIV-1 infected patients who were not lost to follow-up was (median 144 cells/μL, IQR = 66-272).

## Discussion

Khayelitsha Site B TB clinic illustrates the successful integration of public HIV-TB health-care services in South Africa. These services include voluntary counselling and testing of HIV status in >95% of TB patients, drug susceptibility testing on almost all bacteriologic specimens, trimethoprim-sulfamethoxazole chemoprophylaxis in >85% of HIV-1 infected patients, and aggressive ART initiation in HIV-1 infected patients according to national guidelines. Despite these measures, clinical deterioration remains an important clinical entity. During the 24 weeks of follow up, 40% of patients experienced clinical deterioration. In multivariate analyses, significant risk factors for clinical deterioration were HIV-1 infection and a low CD4+ count at tuberculosis diagnosis. Currently, HIV-infected patients present to health care services when they are already profoundly immune-suppressed. This results in a high burden of tuberculosis, which is accompanied by multiple complications during antituberculosis treatment.

A distinct pattern of clinical deterioration emerged during the 24 weeks of follow-up (Figure 3). After 10 weeks of follow-up, we observed a rise in the proportion of patients with a CD4+ count of 200-350 cells/μL who experienced clinical deterioration. Few of these patients had initiated ART (according to national protocol [15]). Further studies are needed to determine whether ART initiated soon after tuberculosis diagnosis in this subgroup could reduce the incidence of clinical deterioration. A triple wave of illnesses occurred in profoundly immune-suppressed HIV-1 infected patients (CD4+ count < 200 cells/μL). The first wave (0-4 weeks) comprised co-morbid illnesses and AIDS-defining illnesses (data not presented). This highlights the profound immune-suppression at tuberculosis diagnosis and the rapid occurrence of AIDS-defining illnesses soon thereafter. The second wave represented co-morbid illnesses, tuberculosis-related illnesses and non AIDS-defining HIV-1 related infections, while the third wave included tuberculosis-related and co-morbid illnesses. This demonstrates that immune restoration during antituberculosis treatment and ART is not without complications [7]. The substantial occurrence of co-morbid illnesses throughout the 24 weeks of follow-up was unexpected. Hepatic, renal and cardiovascular related morbidity and mortality are well described in HIV-infected patients [19]. Similarly, co-morbid illnesses causing death in HIV-1 uninfected tuberculosis patients have also been reported [20]. However, the magnitude of co-morbid illnesses in HIV-1 infected patients receiving antituberculosis treatment has not previously been reported. The plethora of illnesses and the 15 deaths (related to AIDS-defining illnesses, poor adherence, and neurologic TB-IRIS) suggest that the management of tuberculosis in profoundly immune-suppressed HIV-1 infected patients is complicated.

Preventing the transmission of HIV-1 and preserving pathogen-specific immunity in those already infected with HIV-1 (by initiating ART at higher CD4+ counts) will likely reduce the incidence of tuberculosis. The potential benefits include fewer deaths and less difficulty in managing complex drug interactions related to rifampin. However, the massive scale-up of ART to treat patients at higher CD4+ counts would require considerable financial and medical resources. In South Africa, less than 50% of patients who quality for ART under current South African guidelines receive ART[15]. Due to the observational nature of our study, we were unable to compare the incidence of clinical deterioration *after *ART initiation in two groups of tuberculosis patients: those with a CD4+ count of 200-350 cells/μL and those with a CD4+ count < 200 cells/μL. Only patients in the latter group were eligible for ART according to South African guidelines [15].

Fewer deaths than anticipated occurred during the first 8 weeks [5]; many deaths (8 of 17) occurred from weeks 10 to 20 of follow-up. It is possible that patients with severe tuberculosis may have died in the referral hospital, precluding enrolment in our study. Patients requiring hospital admission for fatal AIDS-defining illnesses at tuberculosis diagnosis would similarly have not been enrolled. Ninety-two (32%) of 292 patients were diagnosed with tuberculosis at hospital, suggesting that tuberculosis disease at tuberculosis diagnosis was severe.

Bacterial pneumonia is an important cause of hospitalisation and death in HIV-1 infected patients with [21], or without [22] a diagnosis of tuberculosis. In our clinic-based cohort, only one patient with pneumonia cultured a bacterial organism (*Haemophilus influenzae*). We attribute this finding to the combined antibacterial properties of rifampin and trimethoprim-sulfamethoxazole (TMP-SMX) [23]. Most HIV-1 infected patients (86%) in our study received TMP-SMX chemoprophylaxis (160/800 mg daily).

We diagnosed symptomatic drug-induced hepatitis in one patient. Drug-induced hepatitis, defined as a 5-fold rise in liver enzymes (AST or ALT), is reported in 2-28% of patients receiving antituberculosis treatment [24] and 6% of HIV-1 infected patients receiving both antituberculosis and antiretroviral treatment [25]. In our study, liver function tests were not performed in asymptomatic patients receiving antituberculosis treatment or efavirenz-based ART, according to routine practice. Liver function tests were performed when patients experienced clinical deterioration.

During the course of our study, DST was performed at tuberculosis diagnosis, at 8 and 20 weeks of follow-up and at clinical deterioration. This differs from routine practice in South Africa where only certain patients (such as those receiving retreatment for tuberculosis and those who do not respond to antituberculosis treatment) [14] receive DST due to resource constraints. The ability to perform DST in all patients in our study, regardless of previous tuberculosis, likely expedited diagnosis and appropriate management of MDR-TB, especially where the differential diagnoses included MDR-TB and TB-IRIS.

Our study has some limitations. Our definition of clinical deterioration (symptomatic worsening or failure to stabilise within 24 weeks after initiation of antituberculosis treatment) did not include episodes of clinical deterioration that occurred after 24 weeks of follow-up. Twenty-four weeks of follow-up is a short period of observation. It is possible that the causes for deterioration could differ after 24 weeks of follow-up. The relatively low number of patients diagnosed with MDR-TB during follow-up (n = 5) may underestimate the incidence of MDR-TB presenting after 24 weeks. It is noteworthy that 6 patients were diagnosed with MDR-TB during the 24 weeks of follow-up without fulfilling our definition of clinical deterioration. We have previously described the phenomenon of initial clinical improvement with rifampin-resistant *M. tuberculosis *despite receiving standard antituberculosis treatment [26]. At diagnosis of MDR-TB in these 6 patients, we appropriately intensified their antituberculosis treatment and no subsequent clinical deterioration occurred. We also excluded isolated radiological worsening from our case definition of clinical deterioration. Thus, our finding of clinical deterioration in 40% of patients may be an underestimate. Also, in 14 episodes the cause of deterioration could not be identified. Our study did not evaluate the proportion of patients whose HIV-1 status was known prior to tuberculosis diagnosis. In addition, we included patients initially assessed at the tuberculosis clinic, who subsequently presented to the hospital, and then deteriorated and died during their first hospital admission. Patients who presented to the hospital with fatal tuberculosis may not have been included in the study. A substantial proportion of patients were lost to follow-up (75 of 292 patients); reasons for this require further work. Loss to follow-up did not relate to the degree of immunosuppression of patients: In HIV-1 infected patients, the CD4+ counts of patients who were lost to follow-up were similar to the CD4+ counts of patients who were not lost to follow-up. Finally, this study was conducted at a tuberculosis clinic and a referral hospital that serve a large population with a high incidence of HIV-1-associated tuberculosis; thus, our findings may not be generalisable to other settings.

## Conclusions

The two pandemics of HIV-1 and tuberculosis are inextricably intertwined in Africa. Reducing the extraordinary burden of tuberculosis and clinical deterioration during antituberculosis treatment is a priority for resource-limited settings. Future prospective studies are required to determine (1) the optimal interval from antituberculosis treatment to ART initiation in profoundly immune-suppressed HIV-1 infected patients, and (2) whether ART initiation during antituberculosis treatment at higher CD4+ counts reduces the burden of clinical deterioration.

## Competing interests

The authors declare that they have no competing interests.

## Authors' contributions

DJP, RJW and G. Meintjes were responsible for study conception. DJP, SM, RJW, G. Maartens, HM, VDA, HC, CM, and G. Meintjes were responsible for study design. DJP, SM, SS, JP, G. Meintjes were responsible for data acquisition, DJP, SM, RJW, FB, G. Maartens, HM, VDA, HC, CM, SS, JP, G. Meintjes were responsible for data interpretation. DJP compiled the first draft. SM, RJW, FB, G. Maartens, HM, VDA, HC, CM, SS, JP, and G. Meintjes critically revised the manuscript for important intellectual content. All authors read and approved the final manuscript.

## Disclaimer

The contents of this article are the responsibility of the authors and do not necessarily reflect the views of the US Agency for International Development or the US government.

## Pre-publication history

The pre-publication history for this paper can be accessed here:

http://www.biomedcentral.com/1471-2334/10/83/prepub
